# Stockholders Ohio College Dental Surgery

**Published:** 1852-01

**Authors:** 


Stockholders of the Ohio College of Dental Surgery.—The stockholders will please remember their first regular meeting is to take place on Thursday, 19th of February, 1852, at 10 o'clock, A. M., in the lecture room of the Dental College. It is hoped there will be a full attendance, as there will be much business of importance to transact. Those who cannot possibly be present, should authorize some member to represent them at the meeting.

A constitution and by-laws for their future government will be proposed for adoption. A committee is to be selected for the examination of the next graduating class, and the faculty would like to make report of the manner in which they have discharged the trust confided to them. Much other business of a general nature will be attended to.

